# Culture shock

**DOI:** 10.7554/eLife.33312

**Published:** 2017-12-18

**Authors:** Antoine Molaro, Harmit S Malik

**Affiliations:** 1 Division of Basic SciencesFred Hutchinson Cancer Research CenterSeattleUnited States; 2 Howard Hughes Medical InstituteFred Hutchinson Cancer Research CenterSeattleUnited States

**Keywords:** sex chromsome evolution, unicellularity, multicellularity, cancer cell line, dosage compensation, autosomes, Other

## Abstract

Many different human cell lines, including both normal and cancer cells, appear to converge to a state that contains an unusual number of chromosomes when they are grown in culture.

**Related research article** Xu J, Peng X, Chen Y, ZhangY, Ma Q, Liang L, Carter A, Lu X, Wu C-I. 2017. Free-living human cells reconfigure their chromosomes in the evolution back to uni-cellularity. *eLife* **6**:e28070. doi: 10.7554/eLife.28070

In many organisms, sex is determined by chromosomes. In humans and other animals, for example, females possess two X chromosomes, whereas males have one X and one Y chromosome. This means that females can have two copies of some of the genes that are found on the X chromosome, whereas males have only one copy of these genes. Humans also contain 22 pairs of other chromosomes, known as autosomes, and it is thought that the X and Y chromosomes evolved from these (Bachtrog, 2013; Charlesworth, 1991).

Various organisms have evolved different strategies to cope with the potential dosage imbalance between X and autosomal genes. For example, many female mammals – including humans – deactivate one of their X chromosomes, while some worms down-regulate both X chromosomes (Bachtrog et al., 2014). Some male insects and lizards, on the other hand, are known to up-regulate their X chromosome (Marin et al., 2017). Each of these strategies increases the chances of survival at the level of individuals and species, but is the same true at the level of cells? Now, in eLife, Chung-I Wu, Xuemie Lu and co-workers – including Jin Xu, Xinxin Peng and Yuxin Chen as joint first authors – report that this might not be the case (Xu et al., 2017).

Xu et al. used a technique called SNP genotyping to count the number of autosomes and sex chromosomes in 620 human cell lines derived from normal or cancerous cells that had been grown in the laboratory for varying amounts of time. Normal cells cannot be grown in culture for long periods of time before undergoing senescence, so they must undergo some form of transformation (such as infection with the Epstein-Barr virus) to immortalize them. SNP genotyping can also measure genetic variation by detecting if a cell is homozygous (that is, if the alleles at a given locus are the same) or heterozygous (that is, if the alleles are different). The results showed that, over time, both male and female cells changed into a ‘de-sexualized state’: male cells lost their Y chromosome, while females lost their inactive X chromosome, leaving both with just one X chromosome. However, following the establishment of this de-sexualized state, many of the male and female cells acquired a duplicate of their X chromosome, which resulted in a state with two active X chromosomes (Figure 1).

**Figure 1.**
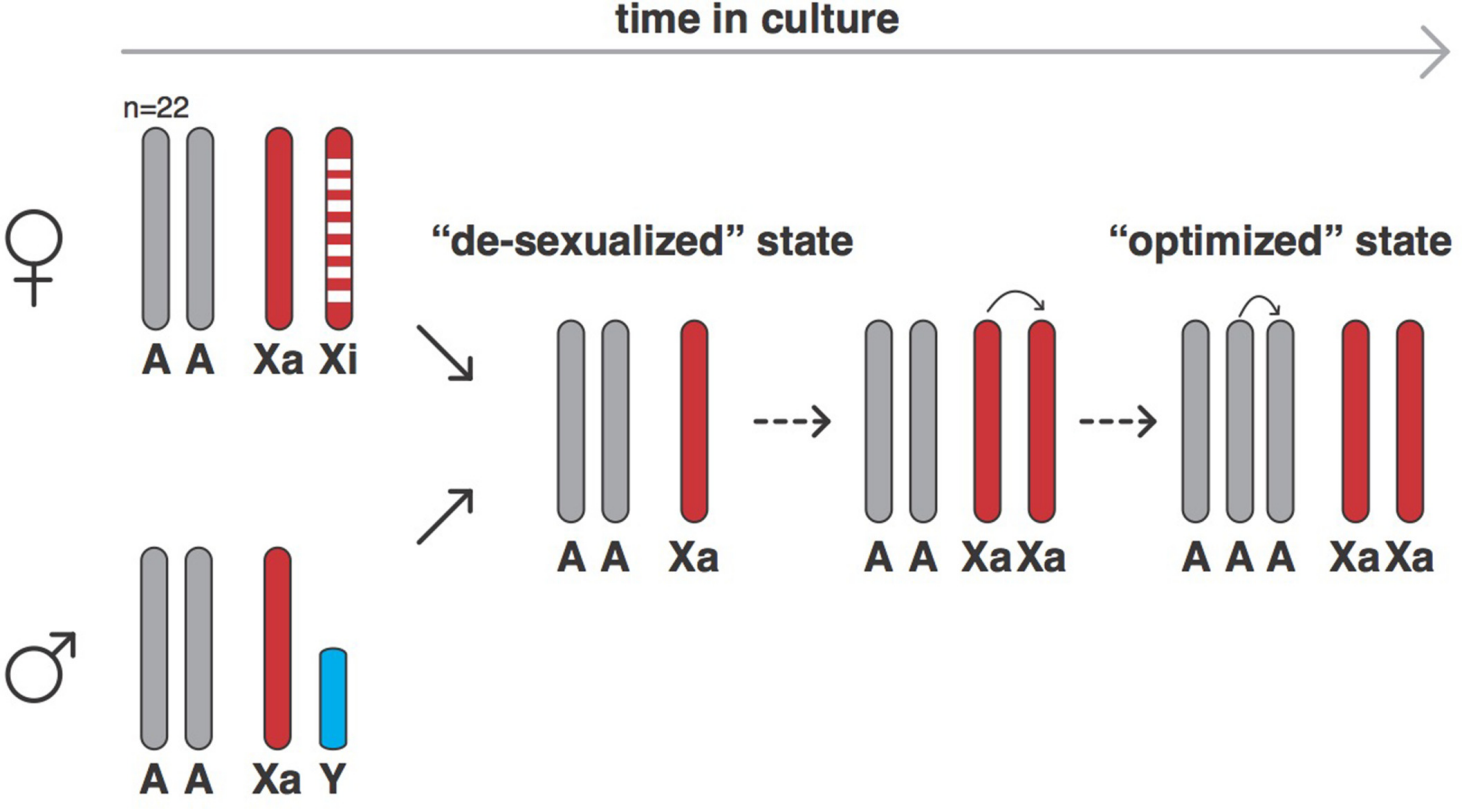
The evolution of human cells in culture. In humans, female cells have 22 pairs of chromosomes, an active X chromosome (Xa) and an inactive X chromosome (Xi), whereas male cells have 22 pairs of chromosomes, an X chromosome and a Y chromosome. Cells derived from human cancers often are aneuploid and contain altered numbers of chromosomes, which have frequently undergone rearrangements from the canonical state. However, when grown in cell culture, both female and male cells (and also both normal and cancer-derived cells) can evolve into a ‘de-sexualized state’ that contains a single X chromosome. In these de-sexualized cells, the X chromosome frequently undergoes a duplication, which is followed by the duplication of one full set of autosomal chromosomes to produce an X:A=2:3 state that contains 68 chromosomes on average (as opposed to 46); this state appears to have been repeatedly selected for in cell culture conditions.

Xu et al. – who are based at institutions in Guangzhou, Beijing, Houston, Stanford and Chicago – then turned their attention to the autosomes and discovered that the cell lines tried to maintain an equal number of all autosomes: in other words, cells tried to have two copies of every autosomal chromosome (an A=2 state) or three copies (A=3). Cells generally evolved from a state with two copies of each autosomal chromosome and two sex chromosomes, to the de-sexualized state which has just one X chromosome and a X:A ratio of 1:2, into a state with a duplicated X chromosome (that is, X:A=2:2) and then, on other occasions, a state in which there are two X chromosomes and three copies of every autosomal chromosome (X:A=2:3; see Figure 1).

As expected the most common X:A ratio was 1:2 but, somewhat surprisingly, X:A=2:3 was the second most common ratio. Xu et al. then modeled their system and found, again to their surprise, that the different ratios were predicted to have different fitness levels. Moreover, the cells were evolving towards a ratio of 2:3, irrespective of how long they had spent in the culture and whether they originally were normal or cancer cells. This probably happens to balance the expression of the genes on the duplicated X chromosomes and the genes on the autosomes.

Xu et al. suggest that the evolution towards a ratio of 2:3 may reflect these cells absolving themselves of some of the functions that cells need to perform in multicellular organisms (Merlo et al., 2006; Chen et al., 2015); for example, if they had remained part of a multicellular organism they would not have been able to jettison superfluous sex chromosomes. The work is particularly interesting because such a unicellular state might lead to the high rates of cell division that are characteristic of cancer.

Evidence for such convergent evolution in cancer cells is growing (Chen and He, 2016; Fortunato et al., 2017). So, just as cancer cells evolve to disobey the constraints of multicellular life (Merlo et al., 2006), the cells in a culture may undergo a form of reverse evolution to a unicellular state (Chen et al., 2015). However, the fact that both normal cells and cancerous cells follow similar rules leaves open the possibility that the preference for the 2:3 ratio is linked to culture conditions, or the transformation method used to immortalize the normal cells, rather than any evolution towards single-celled status.

Regardless of the exact nature of the adaptive challenge that resulted in so many cells displaying the 2:3 ratio, the results of Xu et al. are a great example of a new cellular adaptation that can be studied at the molecular level. These findings also emphasize that even though the X chromosome in human cells used to be an autosome less than 250 million years ago (Graves, 2016), its status in cellular adaptation is distinct from autosomes, and of unique importance to the viability of cells.
